# MicroRNA-20a-mediated loss of autophagy contributes to breast tumorigenesis by promoting genomic damage and instability

**DOI:** 10.1038/onc.2017.193

**Published:** 2017-06-19

**Authors:** L Liu, J He, X Wei, G Wan, Y Lao, W Xu, Z Li, H Hu, Z Hu, X Luo, J Wu, W Xie, Y Zhang, N Xu

**Affiliations:** 1 School of Life Sciences, Tsinghua University, Beijing, China; 2 Key Lab in Healthy Science and Technology, Division of Life Science, Graduate School at Shenzhen, Tsinghua University, Shenzhen, China; 3 School of Pharmacy, Shanghai University of Traditional Chinese Medicine, Shanghai, China; 4 School of Chemical Biology and Biotechnology, Graduate School at Shenzhen, Peking University, Shenzhen, China; 5 Department of Breast Surgery, Peking University Shenzhen Hospital, Shenzhen, China; 6 Department of Clinical Oncology, Wuhan No. 1 Hospital, Wuhan, China; 7 Open FIESTA center, Tsinghua University, Shenzhen, China

## Abstract

Gene expression analysis of The Cancer Genome Atlas (TCGA) breast cancer data set show that miR-20a is upregulated in human breast cancer, especially in triple-negative subtype. Gene Set Enrichment Analysis suggests that miR-20a expression negatively correlates with the autophagy/lysosome pathway. We report here that miR-20a inhibits the basal and nutrient starvation-induced autophagic flux and lysosomal proteolytic activity, increases intracellular reactive oxygen species levels and DNA damage response by targeting several key regulators of autophagy, including BECN1, ATG16L1 and SQSTM1. Re-introduction of exogenous BECN1, ATG16L1 or SQSTM1 reverses the inhibitory effect of miR-20a on autophagy and decreases DNA damage. A negative correlation between miR-20a and its target genes is observed in breast cancer tissues. Lower levels of BECN1, ATG16L1 and SQSTM1 are more common in triple-negative cancers than in other subtypes. High levels of miR-20a also associate with higher frequency of copy-number alterations and DNA mutations in breast cancer patients. Further studies in a xenograft mouse model show that miR-20a promotes tumor initiation and tumor growth. Collectively, these findings suggest that miR-20a-mediated autophagy defect might be a new mechanism underlying the oncogenic function of miRNA during breast tumorigenesis.

## Introduction

Autophagy plays important function in regulating cell homeostasis. Autophagy performs homeostatic ‘quality control’ functions to eliminate unfolded proteins or damaged organelles. Under conditions of high cellular stress, such as during nutrient starvation, bulk autophagy is induced to degrade intracellular components for recycling and building blocks necessary for cell survival.^1^ Dysfunction of autophagy machinery often results in a variety of human diseases, such as cancer.^2, 3, 4, 5^

In the context of cancer, the role of autophagy is controversial and resembles the mythical ‘janus face’. Autophagy plays tumor suppressive function by clearing unfolded proteins or damaged organelles, thereby preventing genome instability.^6, 7, 8^ Loss of autophagy genes perturbs homeostasis and potentially primes cells for tumor development. Many core autophagy genes, including *BECN1*, *UVRAG*, *SH3GLB1 (Bif-1)*, *ATG2B*, *ATG5*, *ATG9B*, *ATG12* and *RAB7A*, are frequently mutated in human cancers.^9^ In various murine models, depletion of essential autophagy genes promotes tumorigenesis. Heterozygous disruption of *BECN1* leads to spontaneous tumor formation in mice, indicating *BECN1* is a halpoinsufficient tumor suppressor,^10, 11, 12^ mice lacking *ATG5*, *ATG7* or *AMBRA1* show similar effect.^13, 14, 15^ However, autophagy is also involved in oncogenic process in some circumstances. Autophagy promotes tumor cell survival and growth within tumor microenvironment, such as hypoxia or metabolic stress. Dysfunction in autophagy pathway suppresses tumor proliferation, dissemination and metastasis.^15^ Inhibition of autophagy increases sensitivity of tumor cells to chemotherapeutic agents as well as radiation therapy.^16, 17, 18^ Deletion of FIP200, a component of the ULK1–ATG13–FIP200 complex that is required for autophagosome formation, suppresses tumor initiation and progression. These findings strongly support the role of autophagy in oncogenesis *in vivo*.^19^

MicroRNAs (miRNAs) contributes to almost all physiological and pathological processes. It is well established that miRNAs control gene expression through a post-transcriptional mechanism that involves mRNA degradation and translation repression.^20, 21, 22, 23^ miRNAs have crucial function in cancer; more than 50% of miRNA genes are located at chromosomal regions that include fragile sites or regions that exhibit genetic deletions or amplifications.^24, 25, 26^ miR-17/92 is the best-characterized oncogenic miRNA cluster.^27^ Human miR-17/92 gene is frequently amplified in solid tumors and several hematopoietic malignancies.^28, 29^ We examined the expression patterns of miR-20a, a member of the miR-17/92 cluster, from TCGA breast cancer data set. A significant upregulation of miR-20a is observed in breast cancer, especially in triple-negative breast cancer. Gene Set Enrichment Analysis (GESA) suggests that miR-20a negatively correlates with the autophagy/lysosome pathway. Studies herein show that miR-20a inhibits basal and nutrient starvation-induced autophagic flux and lysosomal proteolytic activity, increases cellular reactive oxygen species (ROS) levels and DNA damage response by downregulating several key regulators of autophagy, including BECN1, ATG16L1 and SQSTM1. Low levels of BECN1, ATG16L1 and SQSTM1 are predominantly observed in triple-negative cancers than in other subtypes. A large fraction of copy-number-altered genome and DNA mutations are also detected in breast cancer patients with elevated levels of miR-20a. Collectively, our results suggest that miR-20a-mediated dysfunction of autophagy contributes to mammary tumorigenesis by promoting genomic damage and instability.

## Results

### Upregulation of miR-20a in human breast cancer

**Figure 1:**
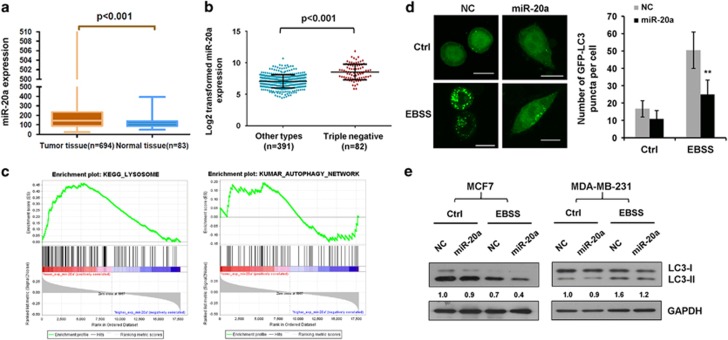
Upregulation of miR-20a in human breast cancer. (**a**) miR-20a expression in normal breast tissues (*n*=83) and breast cancer tissues (*n*=694) in TCGA data set, *P*<0.001 was calculated using nonparametric Mann–Whitney test. (**b**) miR-20a expression is higher in triple-negative breast cancer tissues (*n*=82) than the other subtypes (*n*=391) in TCGA data set, *P*<0.001. (**c**) Gene set enrichment analysis (GESA) comparing the miR-20a lower group (red) against the miR-20a higher expression group (blue) of breast cancer cohorts in TCGA data set, lower expression of miR-20a correlates with autophagy and lysosome pathways. (**d**) GFP-LC3 puncta formation assay in MDA-MB-231 cells overexpressing NC or miR-20a. The samples were cultured in complete medium (Ctrl) or EBSS solution to induce nutrient starvation for 4 h. Scale bar, 10 μm. GFP-LC3 dots per cell was shown as means±s.d. (**P<0.01). (**e**) MDA-MB-231 and MCF7 cells transfected with NC or miR-20a were cultured in normal medium or EBSS solution for 4 h. Relative levels of LC3-II versus GAPDH were analyzed by immunoblotting and Image J software.

Recent studies show that the expression levels of the oncogenic miR-17/92 cluster in triple-negative breast cancer is three times higher than other subtypes.^29, 30^ Here, we analyzed miR-20a expression from TCGA miRNA-seq data set; miR-20a expression was markedly higher in breast cancer tissues (Figure 1a). We identified miR-20a expression signature predictive of estrogen receptor (ER), progesterone receptor (PR) or human epidermal growth factor receptor 2 (HER2). The TCGA samples were subdivided into clinically the relevant subgroups: ER negative (*n*=109), ER positive (*n*=384), PR negative (*n*=164), PR positive (*n*=328), HER2 negative (*n*=411) or HER2 positive (*n*=71) based on immunohistochemical expression of hormone receptors. A significant upregulation of miR-20a expression was observed in ER-negative or PR-negative tumors (Supplementary Figure S1). Tumors with the triple-negative immunophenotyped account for up to 15% of breast cancer. Comparison of miR-20a levels between triple-negative breast cancers (*n*=82) and other subtypes (*n*=391) revealed that miR-20a is upregulated in triple-negative subtype (Figure 1b). These results demonstrate that miR-20a might be a potential diagnostic marker to distinguish triple-negative breast cancer from other subtypes.

### Integrative analysis reveals negative correlation between miR-20a and autophagy/lysosome pathway

Autophagy/lysosome pathway plays important roles in malignant transformation and cancer progression.^15^ To make the connection between miR-20a and the autophagy/lysosome pathway at a molecular level, we performed Gene Set Enrichment Analysis. The expression level of miR-20a was used as a phenotype label to identify biological processes and signaling pathways associate with it. Enrichment analysis of gene expression profiles revealed that expression of certain components of the autophagy/lysosome pathways correlate with lower expression of miR-20a (Figure 1c).

To investigate the role of miR-20a in autophagy, we transfected miR-20a and GFP-LC3 into MDA-MB-231 triple-negative breast cancer cells and cultured cells in Earle's Balanced Salt Solution (EBSS) medium to induce nutrient starvation. miR-20a significantly reduced GFP-LC3 puncta formation in nutrient-starved cells. Immunofluorescence assay with anti-LC3 confirmed these results (Figure 1d and Supplementary Figure S2a). Next, we used two breast cancer cell lines MDA-MB-231 and MCF7 to examine LC3-I to LC3-II conversion. Quantitative real-time PCR showed MDA-MB-231 cells expressed much higher levels of endogenous miR-20a than MCF7 cells, but much lower amounts of miR-20a after transfection, indicating MCF7 cells have much better transfection efficiency than MDA-MB-231 cells (Supplementary Figures S3a and b). miR-20a attenuated LC3-II protein expression in both cells, especially in MCF7 cells presumably because MCF7 cells have higher levels of miR-20a after transfection (Figure 1e).

### miR-20a targets several genes related to autophagy

**Figure 2:**
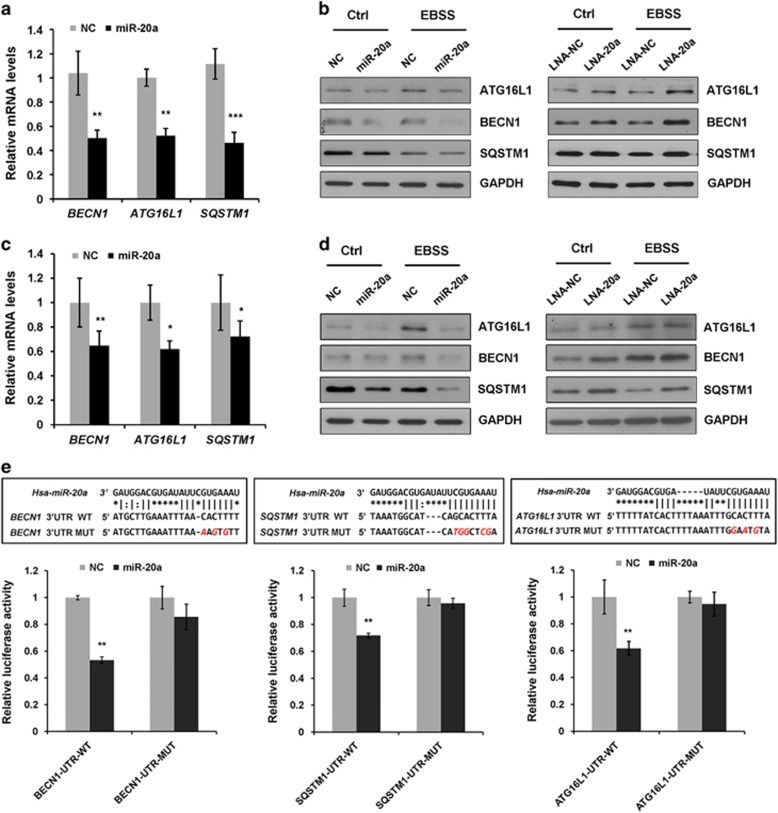
miR-20a targets several autophagy related genes. (**a**) Relative mRNA levels (normalized by GAPDH) of BECN1, SQSTM1, ATG16L1 in MCF7 or (**c**) MDA-MB-231 cells overexpressing NC or miR-20a were analyzed by qPCR. (**b**) MCF7 or (**d**) MDA-MB-231 cells transfected with NC, miR-20a, LNA-NC or LNA-20a were either untreated or treated with EBSS for 4 h. The protein levels of ATG16L1, BECN1 and SQSTM1 were analyzed by immunoblotting. (**e**) Schematic illustration of the predicted miR-20a pairing sites in BECN1, SQSTM1 and ATG16L1 3′UTRs. Luciferase reporters containing wild type (WT) or mutated (MUT) BECN1, SQSTM1 or ATG16L1 3′UTR fragments were co-transfected with NC or miR-20a into MCF7 cells. Relative luciferase activity was normalized to NC (**P<0.01).

We used TargetScan, an miRNA target prediction algorithm, to discover the downstream targets of miR-20a in the autophagy pathway. Several autophagy related genes, including *BECN1*, *ATG16L1* and *SQSTM1* were identified as putative targets. To validate, we performed qPCR and immunoblotting analysis. Overexpression of miR-20a markedly reduced the transcript abundance of *BECN1*, *ATG16L1* and *SQSTM1* in both MDA-MB-231 and MCF7 cells (Figures 2a and c). Immunoblotting revealed that miR-20a suppressed BECN1, ATG16L1 and SQSTM1 protein expression in both cells (Figures 2b and d and Supplementary Figure S4). We were also interested to know whether knockdown of endogenous miR-20a would affect target genes expression. LNA-modified miR-20a inhibitor (LNA-20a) efficiently suppressed endogenous miR-20 expression (Supplementary Figure S3c). LNA-20a moderately increased the amounts of BECN1, ATG16L1 and SQSTM1 in both MDA-MB-231 and MCF7 cells (Figures 2b and d and Supplementary Figure S4).

Next, we employed luciferase assay to examine whether miR-20a directly regulates target genes expression. We cloned the wild-type (WT) 3′-UTRs of *BECN1*, *ATG16L1* or *SQSTM1* gene to a luciferase reporter. In the presence of miR-20a, the relative luciferase activities for *BECN1*, *SQSTM1* or *ATG16L1* wild-type constructs were reduced by 47%, 28% and 39%, respectively. We also disrupted the putative miR-20a paring sites in the 3′-UTRs by site-directed mutagenesis, the mutated reporters (Mut) restored miR-20a-induced inhibition of luciferase activity (Figure 2e). Because BECN1, ATG16L1 and SQSTM1 are key autophagy regulators, we propose that miR-20a may negatively regulate the autophagy/lysosome pathway.

### miR-20a inhibits autophagic flux and lysosomal proteolytic activity

**Figure 3:**
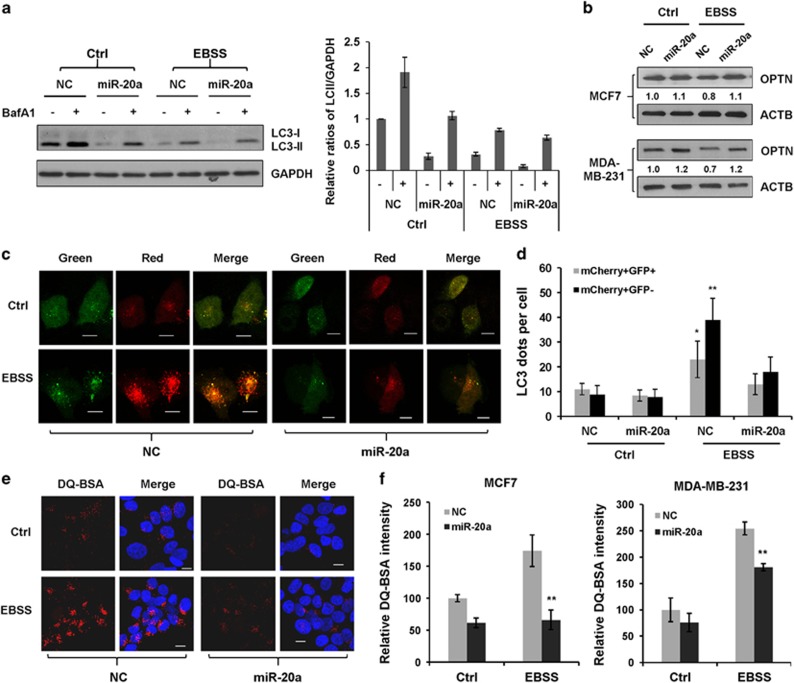
miR-20a inhibits autophagic flux and lysosomal proteolytic activity. (**a**) MCF7 cells transfected with NC or miR-20a were incubated with bafilomycin A1 (Baf A1, 10 nm) for 1 h. The relative amounts of LC3-II versus GAPDH were determined by western blotting and Image J densitometric analysis. (**b**) MDA-MB-231 and MCF7 cells transfected with NC or miR-20a were cultured in normal medium or EBSS solution for 4 h. Protein expression of OPTN and ACTB were detected by immunoblotting. (**c**) Representative fluorescent images of MCF7 cells transfected with miRNAs and mCherry-GFP-LC3. Scale bar, 10 μm. (**d**) Cells with autolysosomes (red dots; mCherry+/GFP−) and autophagosomes (yellow dots; mCherry+/GFP+) were samples from at least 100 cells. (**e**) MCF7 cells overexpressing NC or miR-20a were incubated with DQ-BSA for 12 h, cultured in normal medium or EBSS for 4 h, the fluorescence images of DQ Red BSA were captured by confocal microscopy. Scale bar, 10 μm. (**f**) The red fluorescence intensity of DQ-BSA was quantified with Image J. More than 150 cells were analyzed in each group (**P<0.01).

To investigate the role of miR-20a in regulating the autophagy/lysosome pathway, we used lysosome inhibitors to evaluate autophagic flux.^31, 32^ Immunoblotting revealed that starvation-induced LC3-II turnover was inhibited by bafilomycin A1 (Baf A1) or hydroxychloroquine (HCQ), miR-20a inhibits basal level and starvation-induced LC3-II turnover (Figure 3a and Supplementary Figure S5). Because MCF7 cells express higher amounts of LC3-II and much less visible LC3-I, we performed more assays to precisely measure autophagic flux. SQSTM1/p62 is commonly used to monitor autophagic flux. Because we have shown that miR-20a directly targets SQSTM1, we used OPTN (Optic neuropathy-inducing protein, Optineurin), a most recently identified selective autophagy substrate, to determine autophagic flux.^33^ Nutrient starvation destabilized endogenous OPTN protein, overexpression of miR-20a caused OPTN accumulation, especially in nutrient-starved cells (Figure 3b). Then, we used the tandem mCherry-GFP-LC3 probe to distinguish autolysosomes (mCherry positive/GFP negative; red dots) and autophagosomes (mCherry positive/GFP positive; yellow dots).^34^ Nutrient starvation increased red and yellow LC3 dots per cell; miR-20a-transfected cells showed decreased number of autophgosomes and autolysosomes in nutrient-starved cells, indicating that miR-20a inhibits starvation-induced autophagic flux (Figures 3c and d).

The final stage in autophagy pathway is the maturation of autolysosomes and degradation by lysosomal hydrolases. To visualize and quantify lysosomal proteolytic activity, we employed a fluorescent probe DQ Red BSA.^35^ The probe was nearly undetectable in control cells, dequenching of red fluorescence was observed in cells under nutrient starvation, indicating that starvation stimulates lysosomal activity. Compared with the NC-transfected cells, starvation-induced lysosomal proteolytic activity was markedly reduced in miR-20a-transfected MDA-MB-231 and MCF7 cells. Reduction of lysosomal proteolytic activity was more pronounced in MCF7 cells because MCF7 cells had higher amounts of miR-20a after transfection (Figures 3e and f).

### Blockage of endogenous miR-20a leads to increased autophagic flux

**Figure 4:**
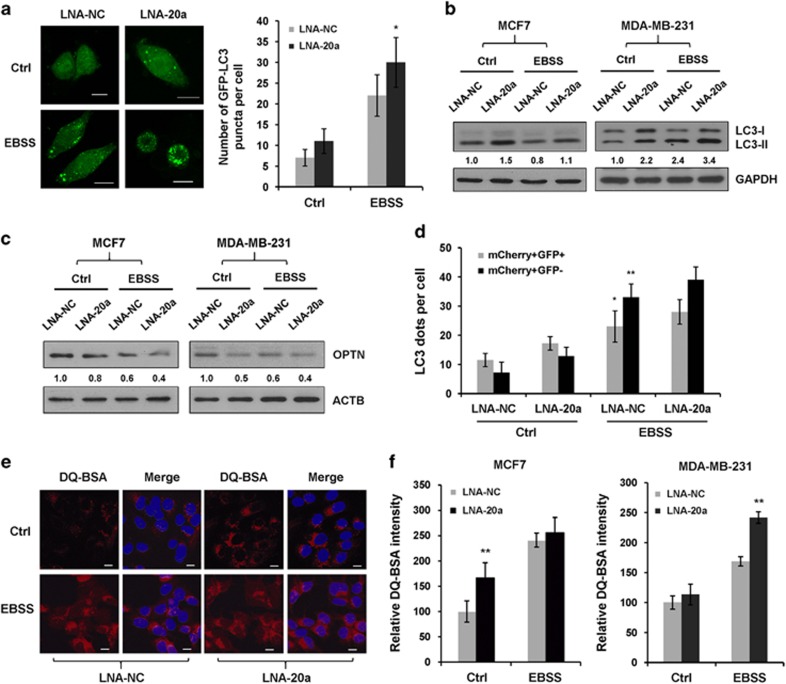
Blockage of endogenous miR-20a promotes autophagy and lysosome activity. (**a**) Representative GFP-LC3 images in MDA-MB-231 cells expressing LNA-NC or LNA-20a. Scale bar, 10 μm. GFP-LC3 dots per cell was shown as means±s.d. (*P<0.05). (**b**) MDA-MB-231 and MCF7 cells transfected with LNA-NC or LNA-20a were cultured in normal medium or EBSS for 4 h. Relative ratios of LC3-II versus GAPDH were assessed by western blotting and Image J software. (**c**) Immunoblotting analysis of OPTN and ACTB in MDA-MB-231 and MCF7 cells transfected with LNA-NC or LNA-20a. (**d**) Quantitative analysis of autophagosomes (yellow dots; mCherry+/GFP+) and autolysosomes (red dots; mCherry+/GFP−) in MCF7 cells transfected with mCherry-GFP-LC3 and LNA-20a or LNA-NC (*P<0.05, **P<0.01). (**e**) Representative images of DQ-BSA in MCF7 cells overexpressing LNA-NC or LNA-20a that subjected with or without nutrient starvation for 4 h. (**f**) Quantification of DQ Red BSA fluorescence in MDA-MB-231 or MCF7 cells (**P<0.01).

To explore the physiological relevance of the above findings, we inhibited endogenous miR-20a expression by LNA-20a. We observed that LNA-20a remarkably increased LC3 puncta formation under nutrient starvation (Figure 4a and Supplementary Figure S2b). Additionally, immunoblotting showed that LNA-20a enhanced the ratios of membrane-bound lipidated LC3-II in both MDA-MB-231 and MCF7 cells (Figure 4b).

Next, we determined whether knockdown of endogenous miR-20a would affect autophagic flux and lysosomal proteolytic activity. In comparison, LNA-20a-transfected cells showed measurable lower levels of OPTN in control and starved cells (Figure 4c). LNA-20a also increased the numbers of autophagosomes and autolysosomes (Figure 4d and Supplementary Figure S6). We used DQ Red BSA to measure proteolytic activity; LNA-20a-transfected cells showed higher levels of fluorescence intensity compared with LNA-NC-transfected cells (Figures 4e and f). These results demonstrate that endogenous miR-20a negatively regulates autophagic flux and lysosomal proteolytic activity.

### miR-20a induces DNA damage response and ROS under nutrient starvation

**Figure 5:**
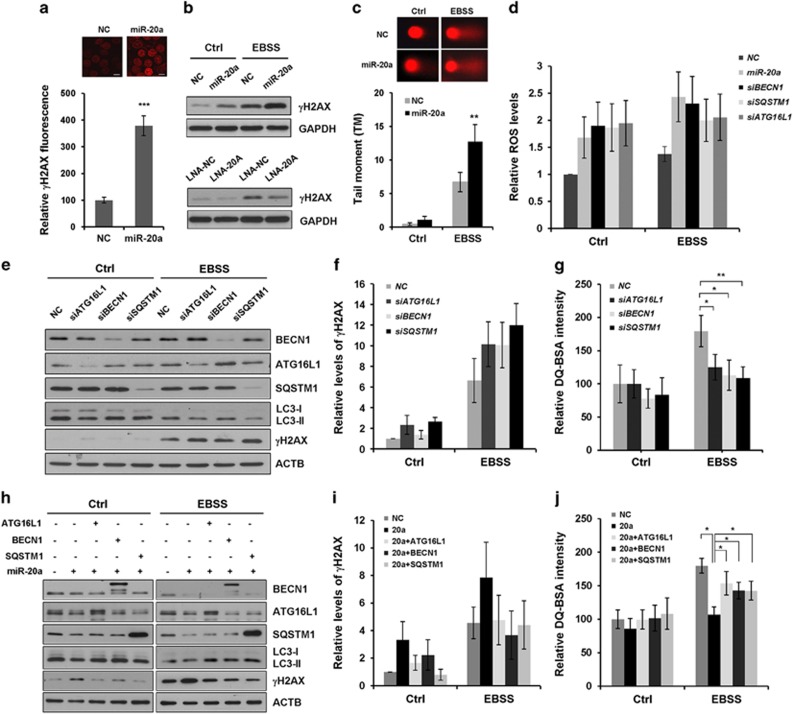
miR-20a increases intracellular ROS levels and DNA damage. (**a**) Immunofluorescence of γH2AX in MCF7 cells overexpressing NC or miR-20a, γH2AX fluorescence intensity was quantified with Image J software (***P<0.001). (**b**) Protein expression of γH2AX and GAPDH were detected by immunoblotting in MCF7 cells overexpressing miRNAs or LNA inhibitors. (**c**) Comet assay in MCF7 cells overexpressing NC or miR-20a. Tail moment (TM) of at least 50 cells in three independent experiments were analyzed by the specific software CASP (**P<0.01). (**d**) MCF7 cells overexpressing miRNAs or siRNAs were cultured in normal medium or EBSS medium for 4 h. Intracellular ROS levels were determined by Amplex Red Hydrogen Peroxide/Peroxidase Assay Kit. (**e**) The expression levels of γH2AX, BECN1, SQSTM1, ATG16L1, LC3 and ACTB in MCF7 cells transfected with siRNAs were determined by western blotting. (**f**) Relative γH2AX protein expression (normalized by ACTB) was determined by Image J densitometric analysis. (**g**) DQ Red BSA fluorescence intensity in MCF7 cells transfected with siRNAs against ATG16L1, BECN1 or SQSTM1 (*P<0.05, **P<0.01). (**h**) miR-20a and plasmids (BECN1-GFP, FLAG-ATG16L1, HA-SQSTM1) were co-transfected into MCF7 cells, cells were cultured in normal medium or EBSS medium for 4 h, samples were collected for immunoblotting analysis. (**i**) Relative γH2AX protein expression (normalized by ACTB) was determined by Image J densitometric analysis. (**j**) Proteolytic activity was analyzed in MCF7 cells expressing miR-20a and plasmids (*P<0.05).

Previous studies have shown that cancer cells with defective autophagic apparatus accumulate DNA damage and deregulated ROS.^36^ We exploited γH2AX, a sensitive marker of DNA damage response, for assessing DNA damage in our system. Overexpression of miR-20a caused distinct γH2AX foci formation in MCF7 cells (Figure 5a). In line with this observation, immunoblotting revealed that miR-20a markedly increased the levels of γH2AX under normal and nutrient-starved conditions, whereas knocking down of endogenous miR-20a showed the opposite effect (Figure 5b). Comet assay and quantification of the tail moment (TM) confirmed that DNA damage was much more pronounced in miR-20a-transfected cells under nutrient starvation (Figure 5c). miR-20a also increased intracellular ROS levels in control and starved cells (Figure 5d).

We examined whether knockdown of miR-20a targets phenocopy the effect of miR-20a on DNA damage and ROS. Immunoblotting revealed that depletion of ATG16L1, BECN1 or SQSTM1 moderately reduced LC3-II levels; DQ Red BSA assay showed that both siRNAs significantly reduced EBSS-induced lysosomal proteolytic activity, indicating that these siRNAs can efficiently block autophagy and lysosomal activity (Figure 5g). Knockdown of ATG16L1, BECN1 or SQSTM1 increased expression of γH2AX and intracellular ROS levels in nutrient-starved cells (Figures 5d–f). These results indicate that defective autophagy increases ROS and activates the DNA damage response.^7^

We also observed whether re-introduction of exogenous BECN1, SQSTM1 or ATG16L1 could provide resistance to miR-20a-mediated autophagy inhibition and prevents DNA damage. Both immunoblotting and DQ Red BSA fluorescence assay showed that overexpression of BECN1, SQSTM1 or ATG16L1 construct was sufficient to rescue miR-20a-induced inhibition of autophagy and lysosomal proteolytic pathway (Figures 5h and j). Additionally, miR-20a*-*induced γH2AX accumulation was markedly reduced by exogenous BECN1, SQSTM1 or ATG16L1 in nutrient-starved cells (Figures 5h and i).

### Upregulation of miR-20a associates with low expression of BECN1, ATG16L1 and SQSTM1 in breast cancer tissues

**Figure 6:**
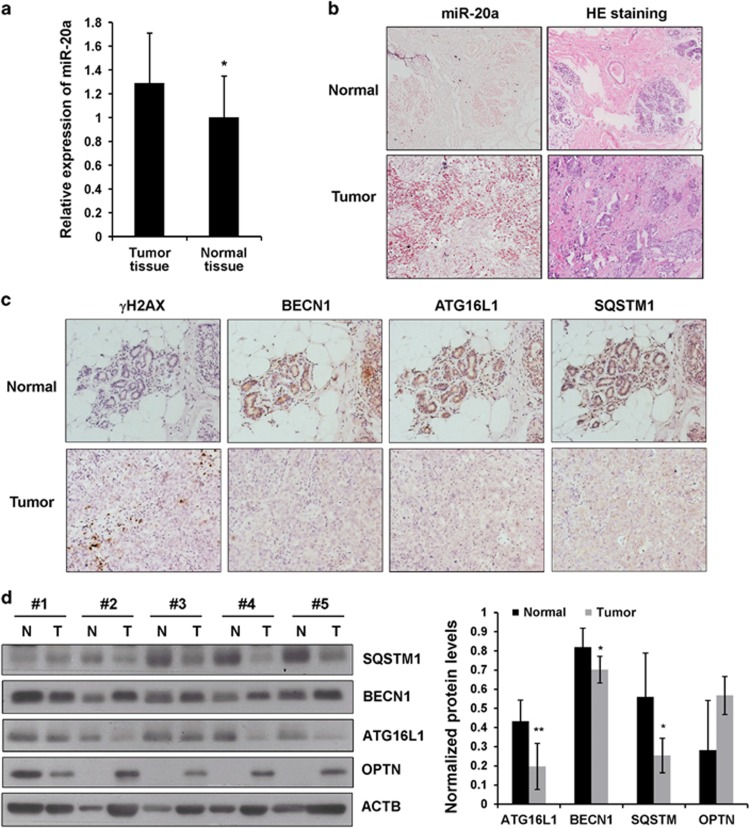
Expression of miR-20a and its target genes in breast cancer specimens. (**a**) Expression of miR-20a in normal breast (*n*=30) and breast tumor tissues (*n*=30) was determined by *in situ* hybridization. (**b**) miRNA *in situ* hybridization and HE staining in tissues from a triple-negative breast cancer patient. (**c**) Immunohistochemical analysis of γH2AX, BECN1, SQSTM1 and ATG16L1 in triple-negative breast cancer tissues and adjacent normal tissues. (**d**) Immunoblotting analysis of SQSTM1, ATG16L1, BECN1 and OPTN in normal mammary tissues and triple-negative breast cancer tissues. Densitometric ratios of BCEN1, SQSTM1, OPTN and ATG16L1 versus ACTB were quantified by Image J software.

Evidence from the above-mentioned *in vitro* experiments indicates that miR-20a suppresses autophagy pathway, induces ROS and DNA damage response. To further define the clinical relevance of our findings, we examined miR-20a expression in normal mammary tissues (*n*=30) and breast cancer tissues (*n*=30) by *in situ* hybridization. Consistent with the TCGA miRNA-seq data set analysis, miR-20a expression was markedly higher in tumor tissues (Figures 6a and b). Immunohistochemical staining of consecutive sections of triple-negative breast cancer specimens revealed that tumor tissues expressed lower levels of BECN1, ATG16L1 and SQSTM1 than normal tissues (Figure 6c). We also performed immunoblotting to explore the expression of BECN1, ATG16L1 and SQSTM1 in tissues from triple-negative subtype. Compared with the normal mammary tissues, breast cancer tissues showed an obvious reduction in BECN1, ATG16L1 and SQSTM1 levels, whereas OPTN expression was higher in cancer tissues (Figure 6d).

**Figure 7:**
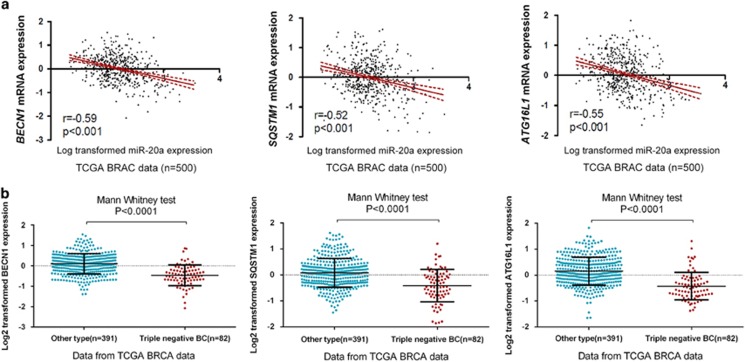
Higher expression of miR-20a is associated with downregulation of *BECN1*, *ATG16L1* and *SQSTM1*. (**a**) Correlation between miR-20a and BECN1, SQSTM1, and ATG16L1 was determined using Spearman coefficient analysis in TCGA breast cancer samples (*n*=500). (**b**) The expression of BECN1, SQSTM1 and ATG16L1 in triple-negative breast cancers (*n*=82) and other subtypes (*n*=391).

We analyzed mRNA expression profiles from TCGA breast cancer cohort (*n*=500). miR-20a expression correlates negatively with that of *BECN1*, *ATG16L1* or *SQSTM1* in breast cancer (Figure 7a). The mRNA expression patterns of *BECN1*, *ATG16L1* or *SQSTM1* were also compared across different breast cancer subtypes. In TCGA data set, the gene expression of *BECN1*, *ATG16L1* or *SQSTM1* were markedly lower in triple-negative breast cancers (*n*=82) compared with other subtypes (*n*=391) (Figure 7b).

### miR-20a increases genomic instability and DNA mutation frequency

**Figure 8:**
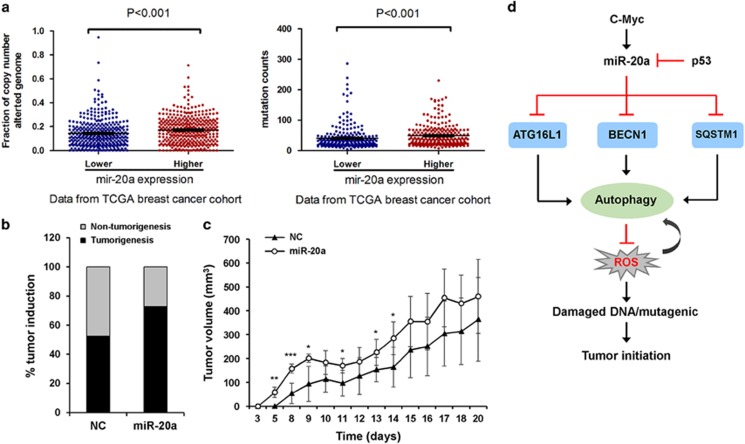
miR-20a promotes tumorigenesis in a xenograft mouse model. (**a**) Genomic instability (fraction of copy-number altered genome and mutation counts) was detected in TCGA breast cancer patients (*P*<0.001, *n*=347). (**b**) MDA-MB-231 cells stably expressing NC or miR-20a were injected into nude mice. miR-20a promotes tumorigenesis compared with NC at day 8 of injection. (**c**) miR-20a promotes tumor growth *in vivo*. Tumor growth curve was determined by measuring the width and length every day (*P<0.05, **P<0.01, ***P<0.001). (**d**) Oncogenic miR-20a directly targets ATG16L1, BECN1 and SQSTM1, inhibits autophagic flux and lysosomal proteolytic activity, increases ROS levels and accumulates DNA damage, which likely contribute to genome instability and tumor-initiating capacity.

Immunohistochemistry showed low levels of miR-20a targets (BECN1, ATG16L1 and SQSTM1) in tumor tissues. However, γH2AX foci formation was dramatically increased in tumor tissues, whereas the staining of γH2AX in normal tissues was nearly undetectable (Figure 6c). In TCGA data set, breast cancer patients with higher levels of miR-20a show higher frequency of copy-number alterations and DNA mutations than cancer patients with lower miR-20a expression (Figure 8a), indicating that miR-20a promotes DNA damage response and increases genomic instability.

### miR-20a promotes tumorigenesis *in vivo*

To investigate the role of miR-20a in tumorigenesis, we generated MDA-MB-231 cell lines stably expressing NC or miR-20a through the lentivirus expression system. Stable transfection of miR-20a markedly suppressed mRNA and protein expression of ATG16L1, SQSTM1 and BECN1 in MDA-MB-231 cells. miR-20a stable transfection also led to reduced LC3-II level and increased OPTN expression, indicating that miR-20a inhibits autophagic flux (Supplementary Figure S7). Nude mice injected with the miR-20a-expressing cells produced more tumors than the mice that were injected with the control cells (Figure 8b). Tumor volume was also significantly larger in mice that were injected with miR-20a-expressing cells (Figure 8c). These results suggest that miR-20a promotes tumor initiation and progression *in vivo*.

## Discussion

Several studies report that the oncogenic miR-17/92 cluster is frequently deregulated in human cancers.^28, 29^ Analysis of miRNA expression profiles revealed a three-fold increase in miR-17/92 expression in triple-negative breast carcinoma. However, comparison of absolute miRNA levels between normal breast tissue and tumor tissues did not show any significant differences.^30^ Here, we analyzed TCGA miRNA-seq data set, upregulation of miR-20a was observed in breast cancer, especially in triple-negative subtype.^37^

The oncogene *c-MYC* directly activates miR-17/92 gene expression. In addition, E2F1 and E2F3 also activate the transcription of miR-17/92 cluster, suggesting that miR-17/92 plays key roles in regulating cell proliferation.^38, 39^ By contrast, the tumor suppressor TP53 transcriptionally represses miR-17/92 expression, through binding to the TATA box of the miR-17/92 promoter.^40^ A recent study report that both two members of the miR-17/92 cluster, miR-20a and miR-17, target the TP53-activating kinase DAPK3, which leads to inactivation of TP53 and upregulation of the miR-17/92 cluster.^41^ Several studies demonstrate that miR-20a promotes proliferation, sustains cell survival and increases angiogenesis by targeting a number of genes, including the E2F family members.^28, 42, 43^ However, in some circumstances, which depends on the physiological context and the cell type, miR-20a plays tumor suppressive function.^44, 45, 46^ Our results show that miR-20a inhibits nutrient starvation-induced autophagic flux. miR-20a suppresses autophagy and lysosomal activity through downregulating the expression of ATG16L1, BECN1 and SQSTM1. Autophagy-deficient cells have deregulated ROS and accumulate DNA damage, which possibly contribute to genome instability and tumor-initiating capacity (Figure 8d).

Autophagy constitutes a barrier to prevent malignant transformation.^15^ Several lines of evidence demonstrate that BECN1 is a haploinsufficient tumor suppressor.^10, 11^ Monoallelic deletion of *BECN1* gene is frequently observed in a high proportion of sporadic breast and ovarian carcinomas.^47^ Low expression of *BECN1* is also found in colorectal, gastric and intracranial tumors.^48^ A recent study reported that *BECN1* expression is reduced in triple-negative breast cancers compared with other subtypes, lower expression of *BECN1* is also associated with poor prognosis.^49^ In line with this report, we demonstrate that miR-20a directly targets BECN1, SQSTM1 and ATG16L1. Upregulation of miR-20a strongly associates with downregulation of BECN1, SQSTM1 and ATG16L1 in human breast cancers, especially in triple-negative subtypes. Therefore, in addition to the monoallelic loss of BECN1 in breast cancer, miR-20a-mediated downregulation of autophagy pathway might be another mechanism that promotes malignant transformation.

Autophagy-deficient cells are impaired in DNA repair by the error-free process of homologous recombination. Cells lacking autophagy machinery largely depend on the non-homologous end joining, the error-prone repair process of DNA double-strand breaks. Sustained reliance on non-homologous end joining results in loss of genetic integrity.^50^ Interestingly, Gene Set Enrichment Analysis analysis indicate that high expression miR-20a is associated with reduction in the levels of proteins involved in DNA repair pathways (data not shown). Thus, we propose that in cancers with higher miR-20a, there is parallel inactivation of autophagy and impairment of DNA repair; this may impair the ability of the cells to adapt to environmental stress, which may subsequently enable gene amplification, mutation and accumulation of DNA damage, thereby promoting genomic instability and cancer progression. Future work should address these questions.

## Materials and methods

### Gene expression analysis

We downloaded TCGA breast cancer data sets from http://tcga-data.nci.nih.gov/tcga/. miRNA-seq analysis was performed from data of 694 breast cancer tissues and 83 normal tissues. RNA-seq analysis was performed from data of 500 breast cancer cohorts. The fraction of copy-number altered genome and mutation counts data were from 347 breast cancer cohorts.

Gene Set Enrichment Analysis (version 2.0) was used to investigate the biological processes that correlate with miR-20a expression in breast cancer as described in the references.^51, 52^ The expression levels of miR-20a were used as phenotype label, and ‘Metric for ranking genes’ was set to Pearson Correlation. All other basic and advanced fields were set to default.

### Cell culture and transfection

MDA-MB-231 and MCF7 cells were purchased from ATCC (Mannassas, VA, USA). MCF7 cells were grown in Dulbecco’s modified Eagle’s medium (Invitrogen, Carlsbad, CA, USA), MDA-MB-231 cells were cultured in ATCC-formulated Leibovitz’s L-15 medium (ATCC), supplemented with 10% fetal bovine serum (PAA Laboratories, Pasching, Austria) and 10 U/ml penicillin/streptomycin (Invitrogen). Cells were cultured in Earle’s Balanced Salt Solution (EBSS; Sigma, St Louis, MO, USA) to induce nutrient starvation.

Lipofectamine 2000 (Invitrogen) was used to transfect miRNAs or siRNAs. miRNAs were purchased from GenePharma Co. Ltd (Shanghai, China). siRNA pools of SQSTM1 (sc-29679) and ATG16L1 (sc-72580) were purchased from Santa Cruz Biotechnology (Dallas, TX, USA). LNA-NC (199020-00) and LNA-20a (426943-00) were purchased from Exiqon (Vedbaek, Denmark). BECN1 siRNA is a pool of two target specific siRNA designed by GenePharm. BECN1 siRNAs: 5′-CAGUUUGGCACAAUCAAUATT-3′ and 5′-AAGAUCCUGGACCGUGUCACCTT3′ Scramble control: 5′-UUCUCCGAACGUGUCACGU-3′.

SQSTM1 plasmid was purchased from Addgene (Cambridge, MA, USA; 28027); BECN1-GFP and FLAG-ATG16L1 plasmids were purchased from GeneChem Co., Ltd (Shanghai, China). Plasmids were transfected into MCF7 cells by Lipofectamine 3000 (Invitrogen).

### Quantitative real-time PCR

mRNA or miRNA expression was determined by quantitative reverse transcriptase–PCR analysis as previously described.^53^ mRNA expression was normalized to GAPDH and miRNA was normalized to U6 snoRNA. The following primer are used for PCR analysis: *GAPDH* forward 5′-AAGGCTGTGGGCAAGG-3′, *GAPDH* reverse 5′-TGGAGGAGTGGGTGTCG-3′ *BECN1* forward 5′-ACCTCAGCCGAAGACTGAAG-3′, *BECN1* reverse 5′-AACAGCGTTTGTAGTTCTGACA-3′ *ATG16L1* forward 5′-AACGCTGTGCAGTTCAGTCC-3′, *Atg16L1* reverse 5′-AGCTGCTAAGAGGTAAGATCCA-3′ *SQSTM1* forward 5′-GATGAGGAAGATCGCCTTGGA-3′, *SQSTM1* reverse 5′-TTCGGATTCTGGCATCTGTAGG-3′.

### Quantitative LC3 puncta analyses

miR-20a and LC3 plasmids (GFP-LC3 or mCherry-GFP-LC3) were co-transfected into MDA-MB-231 or MCF7 cells. Nutrient starvation was induced by treating cells with EBSS for 4 h. Fluorescent images of GFP-LC3 or mCherry-GFP-LC3 were acquired by confocal microscope (FV1000; Olympus, Tokyo, Japan). LC3 dots per cell was calculated from 150 cells in each sample.

### Luciferase assay

The 3′ UTRs fragments of human *BECN1*, *ATG16L1* or *SQSTM1* genes were cloned into pRL-TK luciferase vector (Promega, Madison, WI, USA), the resulting plasmids were called wild-type reporters (WT). To ablate the miR-20 bindings sites, we used site-directed mutagenesis kit (Takara, Kusatsu, Japan) to generate mutated vectors (Mut). MCF7 cells were transfected with luciferase vectors and miR-20a or NC using Lipofectamine 2000 (Invitrogen). Luciferase activity was measured and normalized by protein concentration.

### Western blot

Cells were lysed in ice-cold whole-cell extract buffer as described previously.^53^ Antibodies used are LC3 (L7543, Sigma), ATG16L1 (ab106354, Abcam, Cambridge, UK), BECN1 (#3738, Cell Signaling Technology, Danvers, CA, USA), SQSTM1/p62 (610832, BD Biosciences, Franklin Lakes, NJ, USA), OPTN (A301-831A-T, Bethyl Laboratories, Montgomery, TX, USA), γH2AX (05-636, Millipore, Billerica, MA, USA), GAPDH (Proteintech, Wuhan, China), ACTB (SAB1403520, Sigma), HRP-conjugated secondary antibodies (KPL, Milford, MA, USA). Protein bands were visualized by chemiluminescence (Thermo Scientific, Rockford, IL, USA).

### Immunofluorescence

Cells were fixed in freshly prepared 4% paraformaldehyde solution for 15 min, washed three times with phosphate-buffered saline (PBS) and then treated with 0.25% Triton X-100/PBS for 5 min. The fixed preparations were blocked in 3% BSA/PBS for 1 h, incubated with primary antibodies for 1 h at room temperature, washed three times with PBS and incubated with Alexa Fluor 555 or Alexa Fluor 488 conjugated secondary antibody for another 1 h (Invitrogen). Fluorescent images were acquired by confocal microscopy.

### DQ-BSA staining and quantification

MCF7 cells transfected with miRNAs, siRNAs or LNAs were treated with 10 μg/ml DQ Red BSA (D-12051, Invitrogen) for 12 h at 37 °C. Samples were cultured in normal medium or EBSS solution to induce nutrient starvation. The fluorescent images of DQ Red BSA were acquired by confocal microscopy and the fluorescence intensity was measured by Image J software (National Institute of Health, Bethesda, MD, USA, http://imagej.nih.gov/ij/).

### Comet assay

MCF cells were transfected with NC or miR-20a, cultured in normal medium or EBSS for 24 h. Cells suspension was mixed with low melting agarose (AB0015, Life Technologies, Carlsbad, CA, USA) and plated onto comet slide to perform single-cell gel electrophoresis.^54^ Comet images were captured using a fluorescence microscope (Leica, Wetzlar, Germany); DNA damage was quantified by measuring the Tail Moment (TM) using CASP software (http://casplab.com).

### Determination of ROS

MCF7 cells transfected with miRNAs or siRNAs were incubated in complete medium or EBSS solution for 4 h. Amplex Red Hydrogen Peroxide/Peroxidase Assay Kit (Invitrogen, A22188) was used to analyzing intracellular ROS levels.

### *In situ* hybridization and immunohistochemistry

Paraffin-embedded consecutive slides of normal tissues (*n*=30) and breast cancer tissues (*n*=30) were obtained from Shanghai Outdo Biotech Co., Ltd (Shanghai, China). For *in situ* detection of miR-20a, the slides were hybridized with DIG-labeled oligonucleotide (Exiqon) complementary to miR-20a. Hybridization was visualized by nitroblue tetrazolium/5-bromo-4-chloro-3-indolyl phosphate color substrate (Roche, Basel, Switzerland). The staining intensity of miR-20a was determined using a visual grading system.

For immunohistochemistry, slides were deparaffinized and rehydrated, blocked in sheep serum for 30 min, and then incubated with anti-Beclin 1 (ab55878, Abcam), anti-ATG6L1 (GTX129093, GeneTex, Irvine, CA, USA), anti-SQSTM1 (PM045B, MBL, Woburn, MA, USA) or anti-γH2AX (#9718, Cell Signaling Technologies) overnight at 4°C. The slides were mounted and the images were captured and analyzed by a fluorescence microscope.

### Animal experiment

Four-week-old female BALB/c nude mice were obtained from the Experimental Animal Center of Guangzhou University of Chinese Medicine (Guangzhou, China) and kept in pathogen-free conditions for 1 week. The mice were randomly divided into two groups (*n*=12 for each group). miR-20a or NC was transfected into MDA-MB-231 cells by lentivirus infection. Stable cells were trypsinized and suspended in PBS. Each mouse was subcutaneously injected with approximately 5 × 10^6^/0.2 ml cells at the abdominal region. The tumor size was measured using digital calipers daily for 3 weeks.

### Statistical analyses

All data were expressed as mean±s.d. of three independent experiments. Measurement data between two groups were performed using nonparametric Mann–Whitney test. The correlation of miR-20a and target gene expression was examined by Spearman correlation test. Statistical analyses were performed using two-tailed Student’s *t*-test.
