# Supplementary material for: MicroRNA-20a-mediated loss of autophagy contributes to breast tumorigenesis by promoting genomic damage and instability
Source: Oncogene. 2017 Jun 19;36(42):5874–84. doi: 10.1038/onc.2017.193 (PMC5658668; doi:10.1038/onc.2017.193)
Supplement: Supplementary Figures [file onc2017193x1.ppt]

## Slide 1
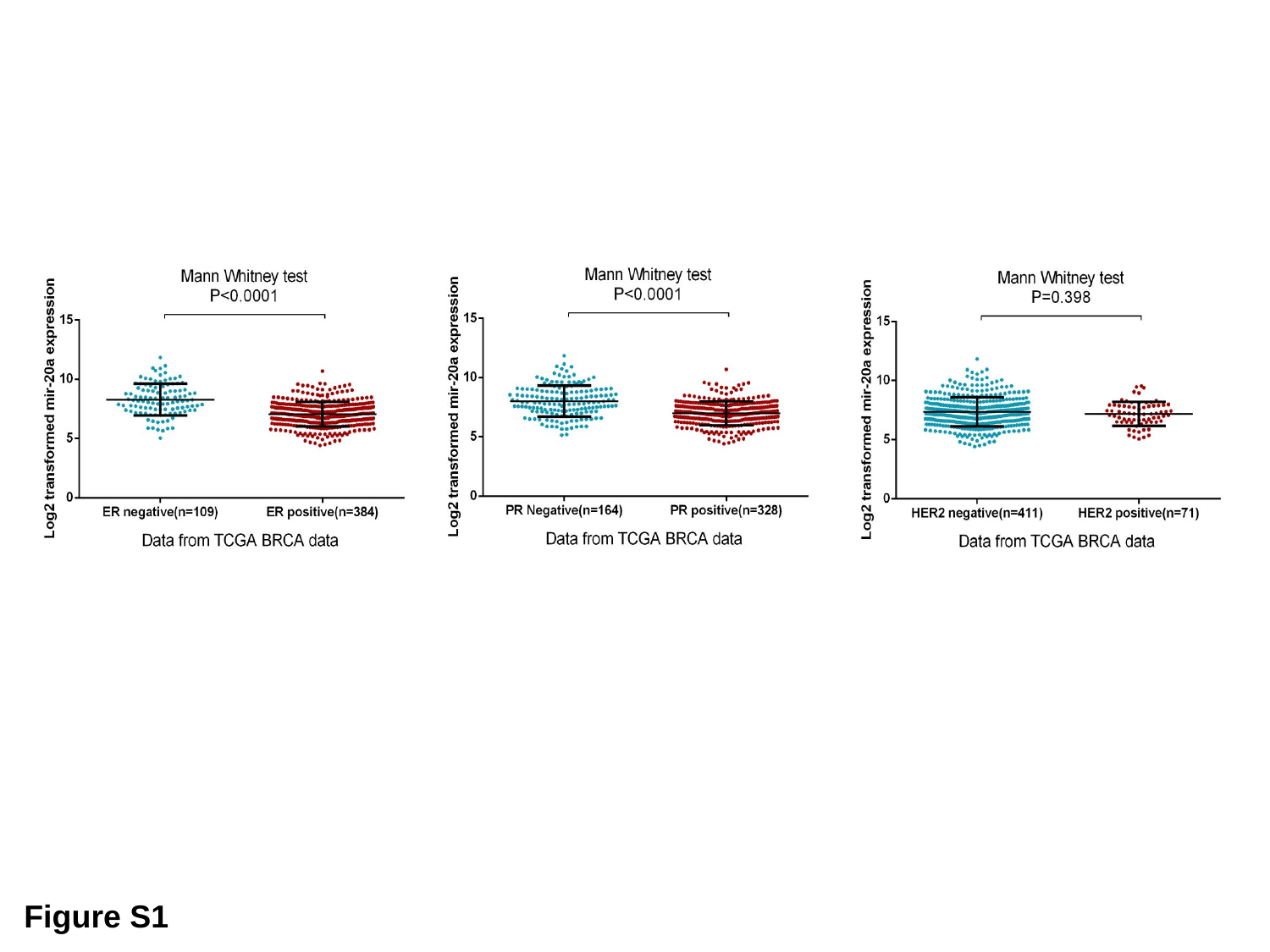

Figure S1

## Slide 2
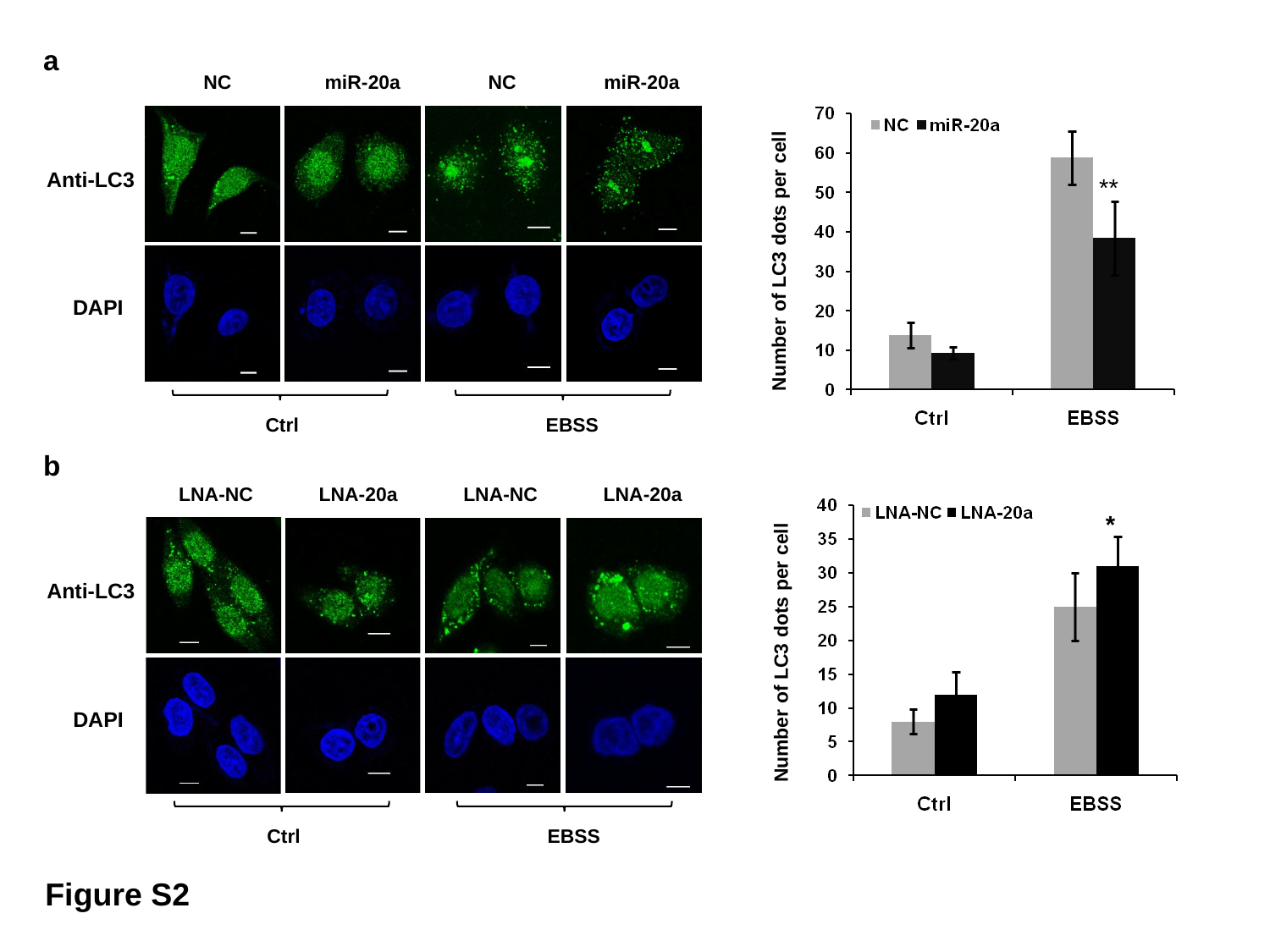

a
b
NC miR-20a NC miR-20a
Anti-LC3
DAPI
 Ctrl EBSS
Number of LC3 dots per cell
**
 LNA-NC LNA-20a LNA-NC LNA-20a
Anti-LC3
DAPI
 Ctrl EBSS
Number of LC3 dots per cell
*
Figure S2

## Slide 3
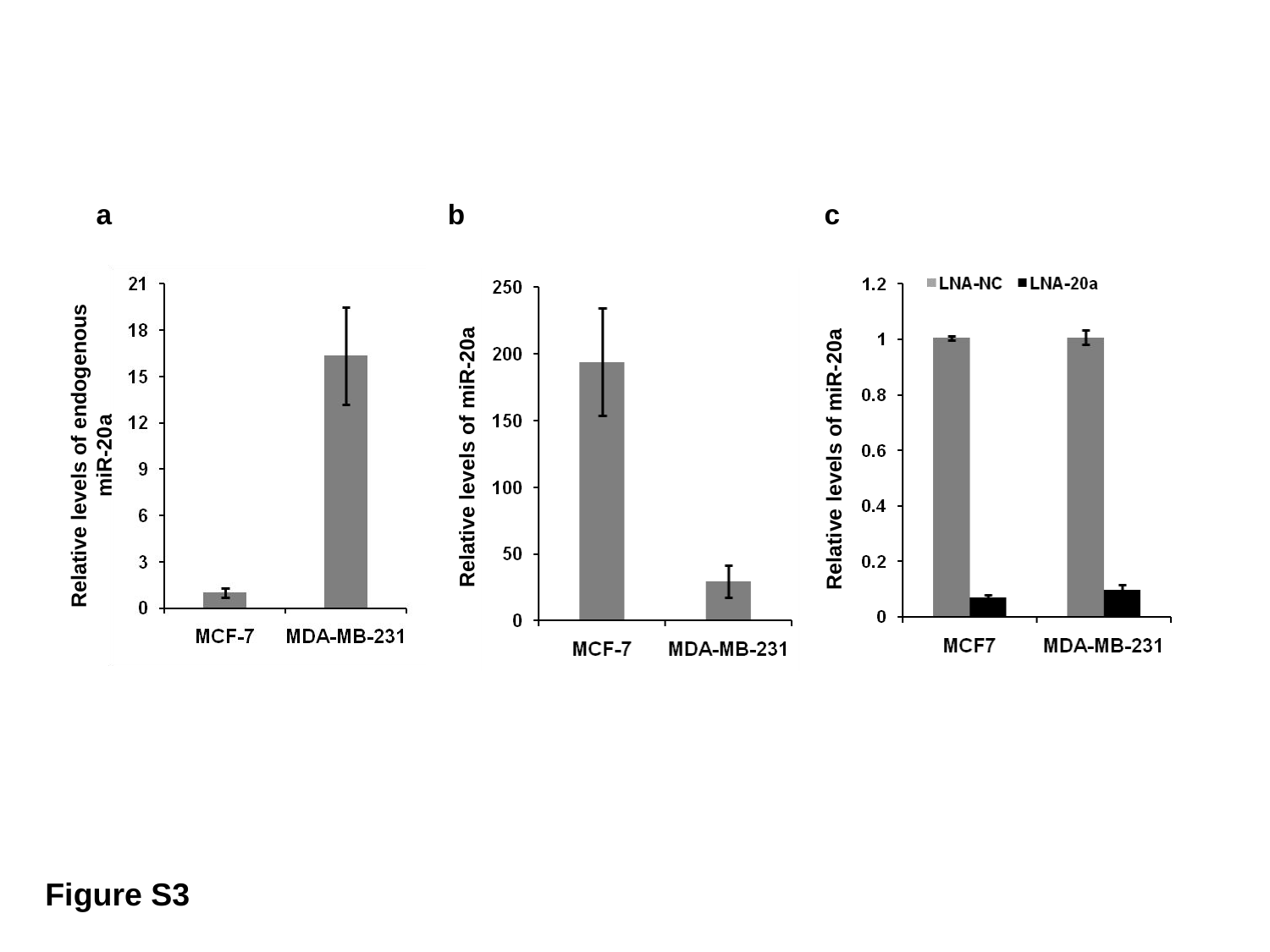

a b c
Relative levels of miR-20a
Relative levels of endogenous
miR-20a
Relative levels of miR-20a
Figure S3

## Slide 4
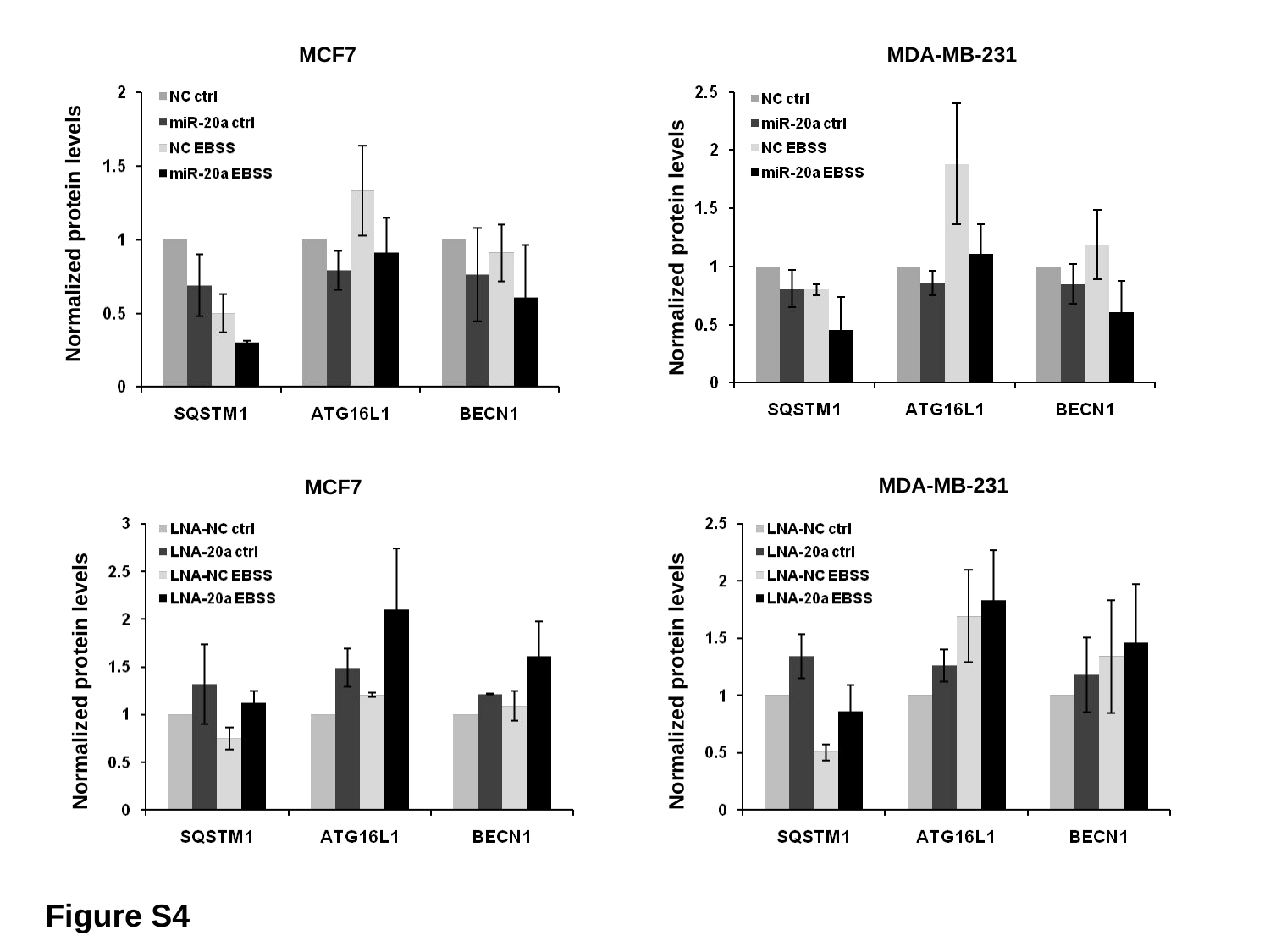

MCF7
Normalized protein levels
MDA-MB-231
Normalized protein levels
MDA-MB-231
Normalized protein levels
MCF7
Normalized protein levels
Figure S4

## Slide 5
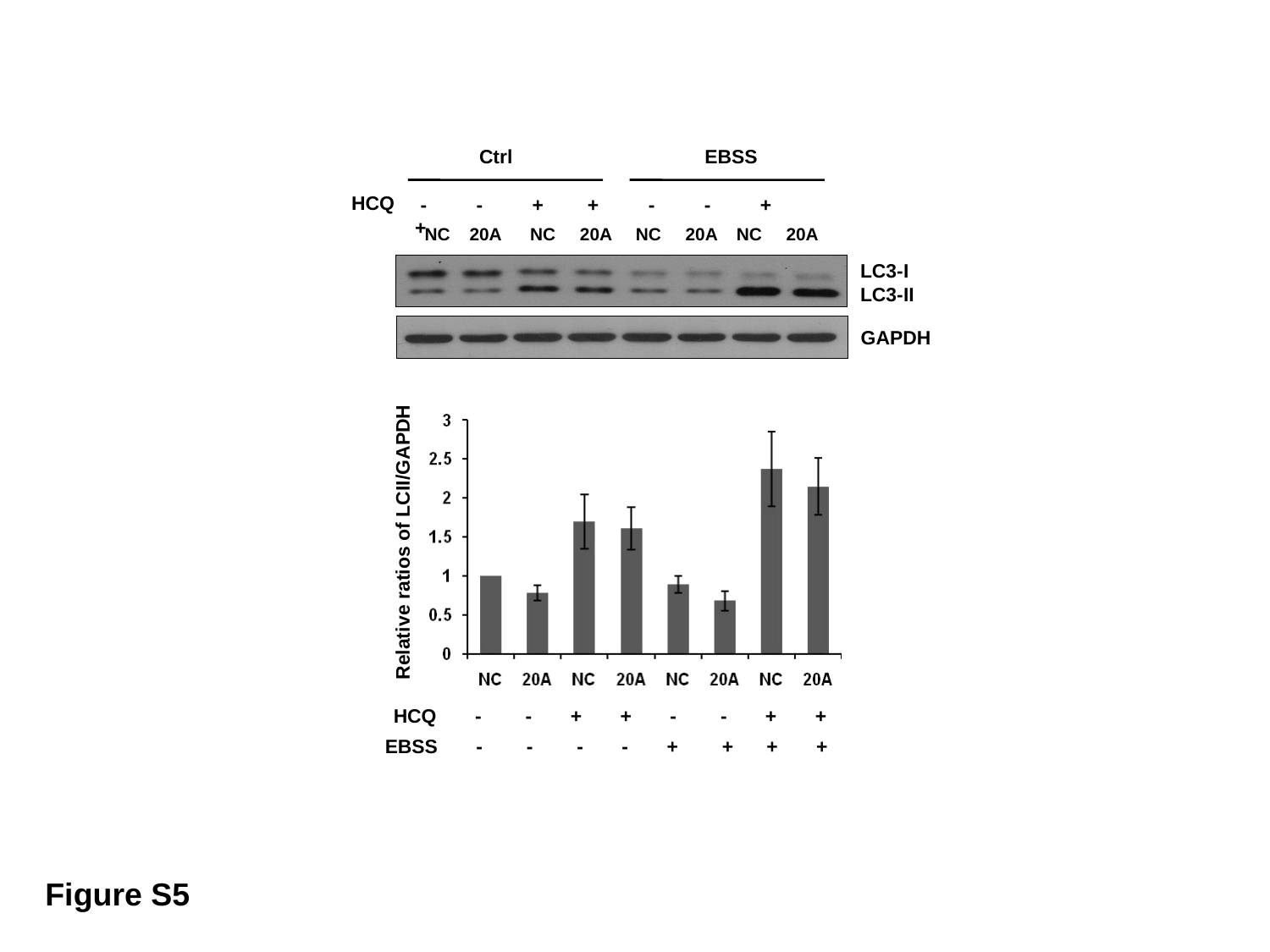

Ctrl EBSS
HCQ
 - - + + - - + +
NC 20A NC 20A NC 20A NC 20A
LC3-I
LC3-II
GAPDH
Relative ratios of LCII/GAPDH
HCQ - - + + - - + +
EBSS - - - - + + + +
Figure S5

## Slide 6
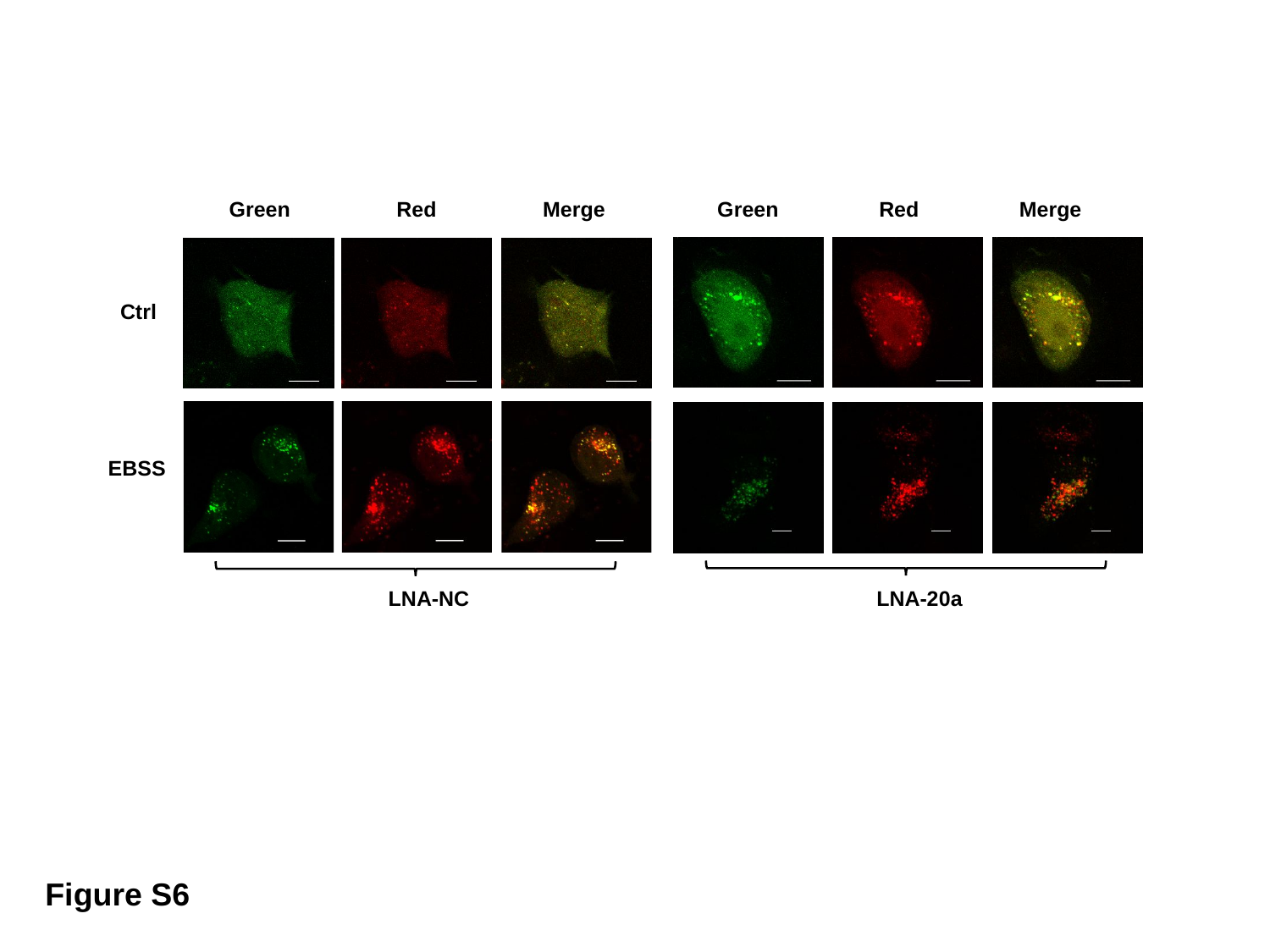

Green Red Merge Green Red Merge
Ctrl
EBSS
 LNA-NC LNA-20a
Figure S6

## Slide 7
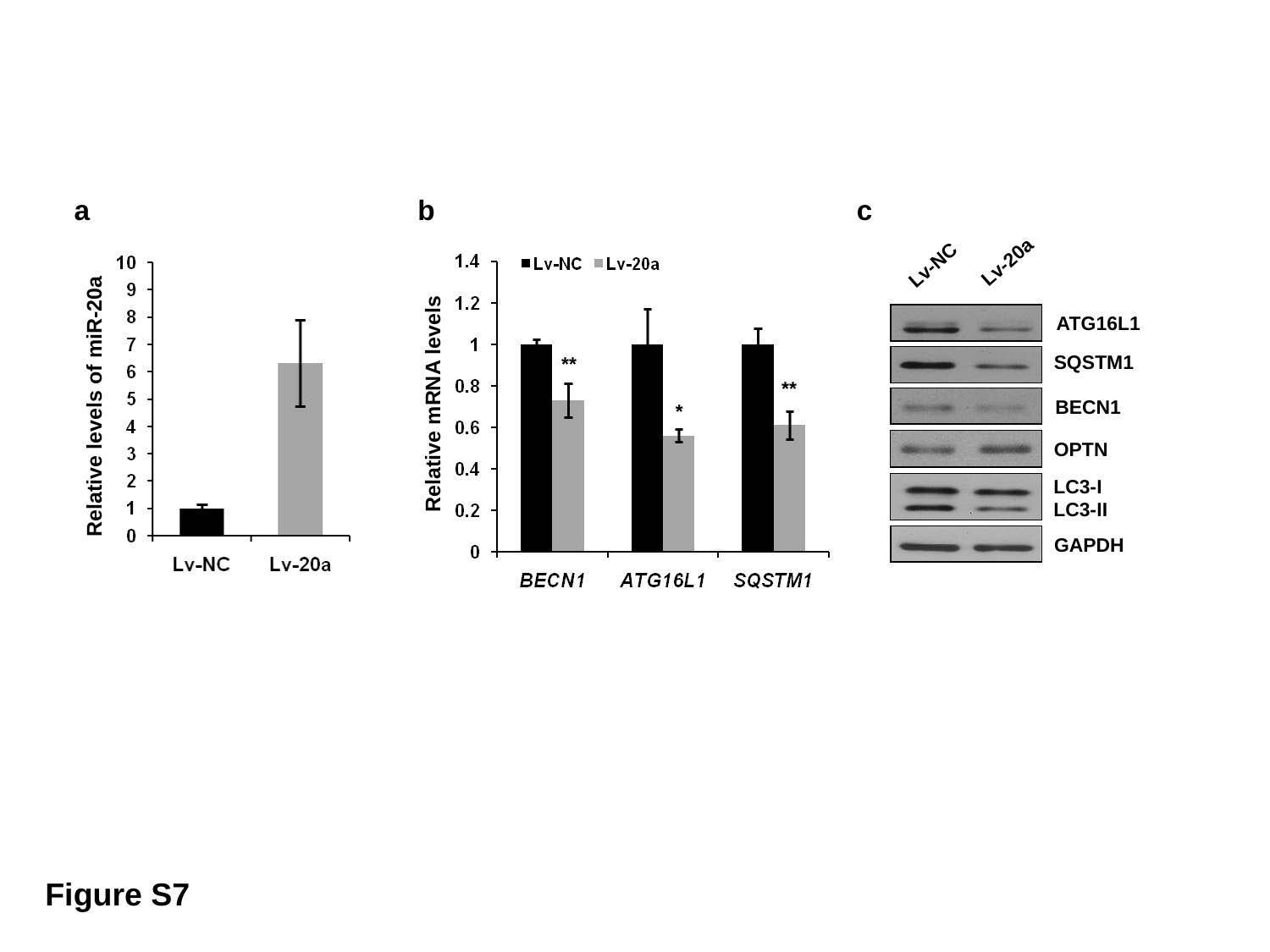

a b c
Lv-20a
Lv-NC
ATG16L1
SQSTM1
BECN1
OPTN
LC3-I
LC3-II
GAPDH
Relative levels of miR-20a
**
**
Relative mRNA levels
*
Figure S7
