# Supplementary material for: MicroRNA-20a-mediated loss of autophagy contributes to breast tumorigenesis by promoting genomic damage and instability
Source: Oncogene. 2017 Jun 19;36(42):5874–84. doi: 10.1038/onc.2017.193 (PMC5658668; doi:10.1038/onc.2017.193)
Supplement: Supplementary Figure Legends [file onc2017193x2.docx]

**Supplementary data**

**Figure legends**

**Figure S1. miR-20a expression and the status of ER, PR or HER2 in human breast cancer.** TCGA breast cancer samples were subdivided into clinically the relevant subgroups: ER positive and negative, PR positive and negative, HER2 positive and negative according to immunohistochemical expression of ER, PR and HER2. Statistical analysis on the expression of miR-20a in ER positive (n=384), ER negative (n=109), PR positive (n=328), PR negative (n=164), HER2 positive (n=71), HER2 negative (n=411) breast cancer patients (*p*<0.001, nonparametric Mann-Whitney test).

**Figure S2. LC3 puncta formation assay.** (**a**) MDA-MB-231 cells transfected with NC, miR-20a, or (**b**) LNA-NC, LNA-20a were treated with or without EBSS for 4 h. Samples were fixed and immunostained with anti-LC3 antibody. Representative images were captured by confocal microscope (Olympus). Scale bar, 10 μm. Quantification the number of GFP-LC3 dots per cell was shown as means ± s.d. (^*^*p*<0.05, ^**^*p*<0.01).

**Figure S3. Quantification of miR-20a expression in MCF7 and MDA-MB-231 cells.** (**a**) Relative expression of endogenous miR-20a in MCF-7 and MDA-MB-231 breast cancer cells was determined by quantitative RT-PCR. (**b**) Transfection efficiency of miR-20a mimmics in MCF-7 and MDA-MB-231 cells, MCF7 cells express much higher levels of miR-20a than MDA-MB-231 cells. (**c**) LNA-20a dramatically suppressed endogenous miR-20a expression in MCF7 and MDA-MB-231 cells.

**Figure S4. Densitometric analysis of miR-20a targets expression.** MCF7 or MDA-MB-231 cells transfected with NC, miR-20a, LNA-NC, or LNA-20a were either untreated or treated with EBSS solution to induce nutrient starvation. Representative immunoblots are shown in Figure 2. Relative protein levels of ATG16L1, SQSTM1, and BECN1were normalized to GAPDH. Data represent the means ± s.d. from three independent experiments.

**Figure S5. miR-20a inhibits EBSS-induced autophagic flux.** MDA-MB-231 cells transfected with miR-20a or NC were treated with HCQ (20 μM) for 1h, samples were collected for immunoblotting. The relative ratios of LC3-II/GAPDH were determined by Image J densitometric analysis.

**Figure S6. Inhibition of endogenous miR-20a promotes autophagic flux.** MCF7 cells were co-transfected with mCherry-GFP-LC3 and LNA-NC or LNA-20a, then cultured in EBSS solution to induce nutrient starvation. Fluorescent images of green or red LC3 puncta were captured by Olympus confocal microscope, scale bar, 10 μM. Quantitative analysis of red and yellow dots were shown in Figure 4d.

**Figure S7. Stable transfection of miR-20a inhibits autophagy and target genes expression.** (**a**) MDA-MB-231 cells were infected with lentiviral particles containing a scrambled RNA (Lv-NC) or miR-20a (Lv-20a) (GeneChem), along with 5 μg/ml polybrene, stable clones were selected with 10 μg/ml puromycin. (**b**) Quantification of mRNA levels of BECN1, SQSTM1, and ATG16L1 in MDA-MB-231 cells stably expressing miR-20a. (**c**) The protein expression of BECN1, ATG16L1, SQSTM1, LC3, OPTN and GAPDH was determined by western blotting.
